# Perinatal Management for a Pregnant Woman with an* MYH9* Disorder

**DOI:** 10.1155/2016/6730174

**Published:** 2016-06-29

**Authors:** Yuka Yamashita, Rei Matsuura, Shinji Kunishima, Yoshie Oikawa, Hirotsugu Ariizumi, Shoko Hamada, Nahoko Shirato, Ryu Matsuoka, Kohichi Ogawa, Akihiko Sekizawa

**Affiliations:** ^1^Department of Obstetrics and Gynecology, Showa University Fujigaoka Hospital, 1-30 Fujigaoka, Aoba-ku, Yokohama 227-8501, Japan; ^2^Department of Advanced Diagnosis, Clinical Research Center, National Hospital Organization Nagoya Medical Center, Nagoya, Japan; ^3^Department of Clinical Laboratory, Showa University Fujigaoka Hospital, Yokohama, Japan; ^4^Department of Hematology, Showa University Fujigaoka Hospital, Yokohama, Japan; ^5^Department of Obstetrics and Gynecology, Showa University School of Medicine, Tokyo, Japan

## Abstract

We diagnosed a primipara woman with an* MYH9* disorder during her pregnancy. A peripheral blood smear with an immunofluorescence analysis is the established method of diagnosing* MYH9* disorders. We provided genetic counseling, as required, which included apprising the woman of the inheritance pattern, the importance of a genetic analysis, and the potential delivery risks for the patient and her offspring. Given that the potential delivery risks are reportedly low, special perinatal management is not necessary for patients with an* MYH9* disorder whose platelet count is above 5.0 × 10^4^/*μ*L, except for rapid blood access.

## 1. Introduction

*MYH9* disorders, whose prototype is May Hegglin Anomaly (MHA), are characterized by a triad of thrombocytopenia, giant platelets, and leukocyte inclusion bodies, to varying degrees, and are occasionally accompanied by nephritis, cataracts, and difficulty hearing [1–3].* MYH9* disorders are rare genetic disorders caused by abnormalities in the nonmuscle myosin heavy chain-9 (*MYH9*) gene located on chromosome 22q12-13 that encodes nonmuscle myosin heavy chain-IIA (NMMHC-IIA). Because* MYH9* disorders are inherited as an autosomal dominant trait, perinatal care providers must care for and monitor not only the patients but also their children. Genetic counseling should include information about the importance of genetic testing, as genetic information can provide epidemiological data, including the rate of complications, and thereby help determine the patient's prognosis [4]. In the present report, we summarize the information that should be included in genetic counseling for pregnant women with an* MYH9* disorder and discuss the key points of perinatal management.

## 2. Case Report

A 35-year-old primipara woman was referred to our university hospital by her primary care physician at 7 weeks of gestation for management of pregnancy and delivery. Her pregnancy was achieved through a natural course. Her mother and grandmother had a history of thrombocytopenia and difficulty hearing, and she also had difficulty hearing. The patient had been advised of low platelet counts, but the cause had never been further investigated. At the 1st trimester, a blood test showed a platelet count of 6.1 × 10^4^/*μ*L. A peripheral blood smear investigation revealed the presence of giant platelets and Döhle-like inclusion bodies in granulocytes. The patient was suspected of having an* MYH9* disorder. Pure tone audiometry showed bilateral sensorineural hearing loss superiority over the treble. Her pregnancy had progressed without trouble except for gestational diabetes mellitus (GDM), which required treatment with a medical diet. We informed the patient and her husband of the characteristics of* MYH9* disorders, which included a 50% probability of their baby inheriting the disease and, for a prenatal diagnosis, we could either perform amniocentesis or percutaneous umbilical cord blood sampling. The patient and her husband chose to avoid the risk inherent with the prenatal diagnosis procedures; we therefore suggested that the baby should be carefully examined after birth.

At 37 weeks of gestation, we performed a Caesarian section under general anesthesia because of breech presentation. We selected general anesthesia because the patient's preoperative platelet count was 6.0 × 10^4^/*μ*L. Based on the British guidelines for the investigation and management of ITP in adults and the international consensus report on the investigation and management of primary immune thrombocytopenia, a platelet count of 8.0 × 10^4^/*μ*L is a reasonably safe count for placing an epidural or initiating spinal anesthesia [5–7]. The total amount of operative bleeding was 867 mL, and a healthy 2760-gram male infant was delivered with Apgar scores of 8 at 1 minute and 8 at 5 minutes after delivery. The results of a neonatal blood test, including platelet count and size and normal leukocyte morphology, and hearing test were normal. Thereafter, the baby was followed up carefully, although he did not have much risk of bleeding according to his phenotype.

**Figure 1:**
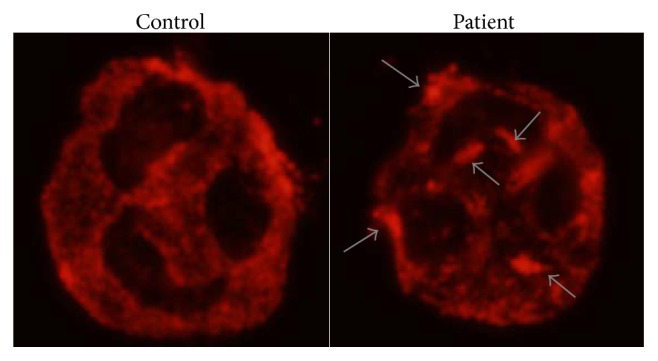
The result of immunofluorescence analysis of peripheral blood smears. The arrow shows abnormal type II aggregation/accumulation of NMMHC-IIA within the cytoplasm of granulocytes.

We also provided the patient with information about the significance of genetic testing, as a genetic mutation can help predict possible complications and the prognosis. An immunofluorescence analysis of peripheral blood smears revealed abnormal type II aggregation/accumulation of NMMHC-IIA within the cytoplasm of granulocytes (Figure 1) [8]. Because the localization pattern of NMMHC-IIA correlates with the site of the* MYH9* mutation and type II localization is closely associated with mutations in* MYH9* exons 26 and 30, these exons were investigated for mutations, and subsequently a heterozygous mutation, p.R1165C, was identified on exon 26. Thus, a diagnosis of an* MYH9* disorder was genetically confirmed.

## 3. Discussion

The perinatal management of pregnant women with* MYH9* disorders consists of an accurate diagnosis (if it has not already been diagnosed), genetic counseling, management of delivery with rapid blood access, and management of complications based on genetic testing.

In the present case, the patient had not been diagnosed with an* MYH9* disorder, so we first had to make an accurate diagnosis.* MYH9* disorders have been shown to account for approximately 30% of congenital macrothrombocytopenia cases worldwide [2, 3], and the actual prevalence of* MYH9* disorders may be higher than that reported before corresponding genetic alterations were identified. For the clinical diagnosis of* MYH9* disorders, a complete blood count (CBC), peripheral blood smear, and assessment of the platelet size and leukocyte morphology on peripheral blood smears are required. Döhle-like inclusion bodies, which are observed in granular leukocytes, are a hallmark of the disease. However, they are sometimes faint and unrecognizable; staining conditions affect the stainability, and the strength of the stain gradually decreases after blood sampling. Conventional May-Grünwald-Giemsa staining is insufficient for the accurate identification of granulocyte inclusion bodies. An immunofluorescence analysis of granulocyte NMMHC-IIA localization is useful for a conclusive diagnosis and classification of* MYH9* disorders [2, 8].

Genetic counseling should include a discussion of the inheritance pattern and what kind of investigations are needed for the patient and the baby; an emphasis on the importance of a genetic analysis; and an explanation of the potential delivery risks for pregnant women with* MYH9* disorders and their babies.* MYH9* disorders are inherited as an autosomal dominant trait. As such, if the mother has an* MYH9* disorder, then her baby has a 50% chance of having the same phenotype. The patient must also be apprised of the importance of the genetic analysis; because a mutation in the* MYH9* gene is closely related to the development of severe complications, a genetic analysis is clinically important for patients [2, 3, 8].

The confirmation of a genetic alteration in the* MYH9* gene leads to an accurate diagnosis and may facilitate the development of a suitable management strategy for the pregnant patient and her baby. If we detect a genetic alteration in the* MYH9* gene of a pregnant woman prenatally, then prenatal assessment of the baby can be performed, via amniocentesis or percutaneous umbilical cord blood sampling. However, because the frequency of severe perinatal complications in such newborns is reportedly very low, even if the baby also has the same phenotype as the mother [1, 9], and because the pregnant patient had no history of severe bleeding, she did not wish to undergo prenatal testing in the present case. We judged that the prenatal management method would not be substantially affected by the outcomes of prenatal testing in this case.

After the delivery, we provided additional genetic counseling and performed genetic testing on the patient. We subsequently detected p.R1165C in the* MYH9* gene, which is often associated with hearing disorders but not nephropathy or cataract [9]. We did not suggest a genetic analysis for the baby, as he did not have any symptoms, such as thrombocytopenia, giant platelets, granulocyte inclusion bodies, or difficulty hearing. In the future, genetic testing of the child may provide further information for the management of any potential future complications. Saposnik et al. said in their report that the platelet count was significantly lower in pediatric patients with bleeding than in those without it. Furthermore, one-third of the pediatric patients showed a bleeding tendency, and 78.9% experienced a bleeding episode before 5 years of age [1].

The appropriate management of delivery is another important problem. Hussein et al. recently conducted a systematic review and reported that 79% of pregnant patients with* MYH9* disorders delivered their babies without hemostatic prophylaxis. Furthermore, a massive postpartum hemorrhage was observed in 5% of pregnancies. Concerning the mode of delivery, vaginal delivery, forceps delivery, and elective Caesarian section were chosen in 55%, 4%, and 24% of cases, respectively, and emergency Caesarian section was required in 15%. Among cases of elective Caesarian section, 12% were performed to avoid a risk of fetal bleeding. The estimated blood loss during delivery was available for 14 pregnancies, and the mean blood loss was 600 mL [9]. Additionally, an examination of the outcomes of four pregnancies showed that three had PPH with* MYH9* disorders [10, 11]. One patient required transfusion in her first and second deliveries, and the others were treated conservatively without hemostatic treatment, emergency uterine artery embolism, or hysterectomy. Concerning infant outcomes, in 78 live neonates, the mean birth weight was 2900 g, and 44% showed similar phenotypes to their mothers. There were no reports of the baby facing severe complications caused by bleeding during the delivery [9]. These findings suggest that pregnant patients should receive perinatal care where blood transfusions are rapidly performed when necessary and that close cooperation among pediatricians, hematologists, nephrologists, and anesthesiologists is critical. If the patient has no history of severe bleeding, vaginal delivery is possible. Furthermore, the baby must be carefully managed. Thus, forceps or vacuum delivery may not be possible in the event of a difficult vaginal delivery [12]. However, Saposnik et al. reported that the number of bleeders was significantly higher when the platelet count was under 5.0 × 10^4^/*μ*L. Measures should be taken to prevent bleeding in patients with a low platelet count before delivery [1]. By extension, if the platelet count is above 5.0 × 10^4^/*μ*L, special delivery management is not necessary for patients with an* MYH9* disorder, except for rapid blood access.
